# The effect of psychosocial interventions by nurses on the well-being of aged Indian adults

**DOI:** 10.6026/9732063002001970

**Published:** 2024-12-31

**Authors:** Gayathri Ellappan Karunakaran, Theranirajan Ethiraj, Shankar Shanmugam Rajendran, Venkatesh Mathan Kumar, Anbalagan Marudan, Palani Pappa Subbiah, Schwartzlin Vinolia Paul Sathyanathan

**Affiliations:** 1 Department of Psychiatric Nursing, College of Nursing, Madras Medical College, Chennai, The TN Dr MGR Medical University, Chennai, Tamil Nadu, India; 2 Department of Paediatrics, Madras Medical College, The TN Dr MGR Medical University, Chennai, Tamil Nadu, India; 3 Department of Paediatric Nursing, College of Nursing, Madras Medical College, The TN Dr MGR Medical University, Chennai, Tamil Nadu, India; 4 Department of Psychiatry, Institute of Mental Health, Madras Medical College, The TN Dr MGR Medical University, Chennai, Tamil Nadu, India; 5 Department of Child Health Nursing, Madras Medical College, The TN Dr MGR Medical University, Chennai, Tamil Nadu, India

**Keywords:** Elderly care, global aging, loneliness, psychosocial interventions, social well-being

## Abstract

The challenge posed by aging, particularly in enhancing psychosocial health in nursing homes has to be addressed. Hence, we evaluated the nurse-led psychosocial interventions to improve social well-being and reduce residents' loneliness. Fifty participants from Chennai engaged in a mixed-methods design, where pre- and post-tests measured social well-being and loneliness. Over four weeks, structured social activities and emotional support interventions resulted in significant improvements in social well-being and notable reductions in loneliness. The study's findings demonstrate that nurse-led psychosocial interventions can effectively enhance well-being and minimise loneliness in elderly care settings, supporting the need for targeted strategies in elderly care.

## Background:

The worldwide population is experiencing unparalleled rapid aging [1]. According to the World Health Organisation, the percentage of the global population aged 60 and beyond is projected to almost double from 12% in 2015 to 22% in 2050 [2]. This demographic transition presents substantial obstacles for healthcare systems, requiring a rise in comprehensive strategies that cater to the diverse requirements of older people [3]. With advancing age, persons frequently encounter a multitude of psychosocial obstacles that might impact their mental and social welfare [4]. Diminished physical well-being, bereavement and the shift to assisted living settings might result in social isolation and manifestations of depression. Increasingly, the psychological well-being of older persons is seen as a crucial element of general health, impacting quality of life and even physical health results [5]. The level of social well-being, which refers to the degree to which individuals have a feeling of belonging and inclusion, possess a well-defined network of family and friends and participate in fulfilling social activities, is especially crucial [6]. Evidence suggests that increased social well-being can result in more significant mental health, decreased likelihood of illness and death and higher cognitive abilities [7]. An investigation revealed that elderly individuals with strong social relationships had a 50% higher probability of survival throughout a specific time than those with weak social bonds [8]. The presence of elderly homes for seniors presents a valuable chance to adopt focused interventions that can improve their health and well-being. Wellness experiences in these environments encompass physical health regimens and programs designed to enhance mental and emotional well-being through social engagement and community-building activities [9]. Nurses are crucial in providing healthcare services for elderly individuals, particularly in environments such as old homes [10]. Frequently, they serve as the leading providers of care and possess a distinct advantage in shaping the everyday experiences of their residents. Evidence suggests that nurse-led psychosocial interventions, such as structured social activities, personalised emotional support and community involvement techniques, can improve social well-being and general health. These treatments operate on the assumption that nursing personnel can provide individualised, uniform psychosocial care that precisely addresses the unique requirements of every resident [11]. Therefore, it is of interest to address these deficiencies using a mixed-methods methodology to thoroughly assess the efficacy of nurse-led psychosocial interventions in improving the social well-being and wellness experiences of elderly individuals living in nursing homes.

## Methodology:

This study employed a mixed-method approach, an explanatory sequential design, to evaluate the effects of nurse-led psychosocial interventions on the social well-being and wellness experiences of elderly individuals residing in a nursing home. Using a one-group pre-test and post-test design, the quantitative component assessed changes in social well-being in a specific nursing home in Chennai. Participation in this phase was limited to fifty elderly individuals. Data were gathered using the De Jong Grieved Scale to measure loneliness and the Multidimensional Scale to measure perceived social support. The qualitative component utilises a phenomenological methodology to thoroughly investigate the wellness experiences of six residents using unstructured interviews. This facilitated gathering detailed and vivid narratives of the residents' experiences before and after the intervention. Both research components were carried out over four weeks. The intervention included organised activities such as gentle physical exercise, strolling, meditation, arts and crafts and group talks to improve relationships and emotional well-being. The quantitative phase data were analyzed using SPSS version 22. McNemar's test and paired t-tests were employed to establish statistical significance, while thematic analysis was used to explain the qualitative data. The study obtained clearance from the Institutional Ethics Committee of Madras Medical College, Chennai, to comply with ethical norms.

## Results:

## Demographic data of the participants:

The study predominantly involved female participants (92%), primarily within the age range of 61-70 years, evenly split between 61-65 and 66-70 age brackets (36% each). Educational attainment was generally low, with 30% having completed primary education and 22% holding a high school diploma. A significant majority identified as Hindu (96%) and were widows (66%). Approximately 44% of the participants had two children and 60% classified their primary source of income under "Others," while 30% reported having no income. Most participants had been residing in their current living arrangement for 1-2 years (66%) and lived alone (96%). The prevalent health issues were hypertension (26%) and diabetes mellitus (22%).

## Pre-test level of social well-being:

Initial findings indicate that 80% of the individuals displayed poor levels of social well-being, while just 20% demonstrated a moderate degree. Not a single participant had high levels of social well-being, suggesting notable early difficulties in this domain (Table 1).

## Effectiveness of nurse-led psychosocial intervention:

The nurse-led psychosocial intervention substantially affected the participants' social well-being, as evidenced by the mean score rising from 21.78 (25.93% of the most significant achievable score) to 43.82 (52.17% of the highest possible score). This 26.24% increase signifies a considerable improvement in social well-being. Furthermore, the scores measuring loneliness showed a substantial decline from 9.10 (82.73%) to 5.30 (48.18%), indicating a 34.55% decrease in loneliness levels after the session (Figure 1 - see PDF).

## Association of post-test level of social well-being:

The examination of post-intervention data showed that widows and pensioners had superior social well-being scores compared to other demographic groups. By contrast, individuals in the age group of 71-75 and those who had lived in the nursing home for a period of 2-5 years indicated more significant levels of loneliness. The results emphasize the impact of marital status, source of income, age and length of stay on older adults' social and emotional health outcomes.

## Exploring the wellness experience:

Qualitative analyses yielded insights into three main themes affecting social well-being: "Comparison to Home," "Distress and Domination," and "Socialization." Participants expressed strong emotional ties and nostalgia under the "Comparison to Home" theme, while "Distress and Domination" revealed challenges related to power dynamics within the nursing home. The "Socialization" theme emphasized the impact of social interactions, environmental quality and care on participants' social integration and overall well-being.

## Integration of quantitative and qualitative findings:

Integrating quantitative and qualitative findings painted a comprehensive picture of the social well-being and loneliness among older adults. Initial assessments showed a low social well-being mean score of 21.78, which improved post-intervention to 43.82. Similarly, loneliness decreased significantly from a pre-test mean of 9.10 to a 5.30 post-test. The qualitative themes provided a deeper understanding of the factors influencing these changes, such as emotional connections, social interactions and internal power structures within the living environment, highlighting the necessity and effectiveness of targeted psychosocial interventions.

This research advances understanding by highlighting the unique role of nurse-led psychosocial interventions in improving older adults' multidimensional well-being. Using a mixed-methods approach, it combines measurable outcomes with personal insights to provide a comprehensive evaluation. The findings offer practical implications for integrating cost-effective, nurse-led interventions into healthcare policies and practices.

## Discussion:

The present investigation systematically assessed the pre-test levels of social well-being among elderly individuals in a nursing home environment, uncovering a significant occurrence of low social well-being, with 80% of participants first categorized in this group. This result is consistent with the previous study by Saha *et al.* However, our study showed a slightly more significant percentage of individuals with impaired well-being, maybe due to differences in social settings or evaluation methods [12]. Similarly, Srivastava *et al.* documented a somewhat more positive initial condition, indicating that geographical or cultural variations could impact the social well-being of elderly populations [13]. Substantial enhancements in both social well-being and loneliness levels revealed the efficacy of the nurse-led psychosocial intervention. The significant impact of focused therapies is seen in the rise in social well-being scores from 25.93% to 52.17%, the highest achievable score and the decrease in loneliness from 82.73% to 48.18%. The effects shown in this study are significantly better than those documented by Kwan *et al.* perhaps due to variations in the intensity or duration of the interventions implemented. Moreover, the results align with the investigations conducted by Wong *et al.* [14], emphasizing the capacity of nurse-led programs to improve feelings of isolation and increase social interaction among older adults [15]. Furthermore, our study revealed a noteworthy correlation between specific demographic factors and the levels of social well-being seen after the test. Specifically, we emphasized the impact of marital status, source of income and length of residency on the emotional and social health of elderly individuals. This discovery is corroborated by the research conducted by Kasa *et al.* which indicated that widowed persons may experience more advantages from muscular social support systems than their unmarried counterparts [11]. In contrast, the divergence in results with Naik *et al.* concerning the influence of age on loneliness underscores the possible diversity in social conventions and support structures among various contexts [16]. The use of qualitative methods in this study enhanced our comprehension of the social well-being of older persons by revealing significant themes such as "Comparison to Home," "Distress and Domination" and "Socialisation." The topics above exemplify the intricate interaction of emotional bonds, power relations and social contacts within the setting of a nursing home. Shin *et al.* [17] and Al-Ghafri *et al.* [18] investigated similar topics, providing evidence for the substantial influence of family relationships and social interactions on the emotional well-being of older adults. Quantitative and qualitative data synthesis thoroughly examines the elements that impact social well-being and feelings of isolation among elderly individuals. The significant changes seen after the intervention confirm the efficacy of the psychosocial techniques used and emphasize the need to consider environmental and interpersonal factors when developing interventions for this group. These findings are supported by research conducted by Cassandra *et al.* [19] and Lindsay-Smith *et al.* [20], which highlighted the significance of environmental and emotional support in improving the well-being of elderly individuals. This study underscores the critical need for targeted psychosocial interventions to address the low levels of social well-being and high levels of loneliness among older adults in residential care settings. By integrating quantitative and qualitative research methods, we provided compelling evidence of the efficacy of nurse-led interventions. We highlighted the complex factors that influence older adults' social health. Future research should explore additional demographic and psychosocial variables to refine intervention strategies further and optimize outcomes for this vulnerable population.

## Conclusion:

The efficacy of nurse-led psychosocial interventions in augmenting social well-being and mitigating feelings of loneliness among elderly individuals residing in nursing homes is reported. Quantitative evaluations with qualitative observations offer a sophisticated comprehension of how psychosocial well-being may be enhanced through focused approaches that effectively tackle environmental and interpersonal elements. The notable enhancements in social well-being and reduction of loneliness underscore the crucial influence of customized treatments in enhancing the quality of life for older people. Future studies should prioritize expanding their implementation to a wide range of situations and people to optimize the impact and application of these interventions.

## Figures and Tables

**Table 1:** Pre-test level of Social well-being

**Level of older adults**	
**Social wellbeing score**	**N**	**%**
Low	40	80.00%
Moderate	10	20.00%
High	0	0.00%
Total	50	100%
